# Affecting Length of Stay in Well-appearing Febrile Infants

**DOI:** 10.1097/pq9.0000000000000359

**Published:** 2020-10-23

**Authors:** Madeline Mier, James W. Antoon, Sarah Sefcovic, Seema Awatramani, Andrew Kreppel, Sara Boblick Smith

**Affiliations:** From the * Division of Hospital Medicine, Department of Pediatrics, Johns Hopkins All Children’s Hospital, St Petersburg, Fla.; † Monroe Carell Jr. Children’s Hospital at Vanderbilt, Nashville, Tenn.; ‡ Division of Hospital Medicine, Department of Pediatrics, Vanderbilt University School of Medicine, Nashville, Tenn.; § Department of Pediatrics, St Anthony’s Hospital, Chicago, Ill.; ¶ UrgiKids Pediatric Urgent Care, Naperville, Ill.; ∥ Department of Pediatric and Adolescent Medicine, University of Illinois Hospital and Health Sciences System, Chicago, Ill.

## Abstract

The management of infants under 2 months of age presenting with fever has perplexed pediatricians for decades. The University of Illinois at Chicago was selected as one of the primary sites for the REVISE (Reducing Variation in Infant Sepsis Evaluations) study through the American Academy of Pediatrics. Our primary objective was to decrease the length of stay (LOS) for well-appearing febrile infants by 20% over 8 months from December 2016 to August 2017.

**Methods:**

We introduced the use of a decision support smartphone application to providers caring for febrile infants. Monthly retrospective chart review of patients 7–59 days old with fever seen in the emergency department or the inpatient setting was performed from September 2015 to August 2016 for baseline data, from December 2016 to August 2017 for intervention data, and from September 2017 to December 2018 for surveillance data.

**Results:**

A total of 1013 patients of ages 7–59 days seen in the emergency department or inpatient unit between September 2015 to December 2018 were screened for study inclusion. Forty-one febrile, well-appearing infants of ages 7–59 days met inclusion criteria. During the baseline period, there was a mean LOS of 48 hours. Intervention and surveillance data did not change the mean from baseline.

**Conclusions:**

Infants with a negative diagnostic evaluation for urinary tract infection, bacteremia, or meningitis drove our LOS. Further study is needed to affect the LOS in febrile infants with diagnoses of urinary tract infection, bacteremia, or meningitis.

## INTRODUCTION

The acute management of infants under 2 months of age presenting with fever has perplexed pediatricians for decades.^1,2^ The appropriate amount of diagnostic testing and clinical observation to determine conclusively whether or not the infant has a severe bacterial infection is under debate, because the utilization of resources for increasingly rare conditions is significant. Despite a growing body of literature over the last 40 years, the evaluation of low-risk febrile infants remains highly variable across children’s hospitals within the United States.^3^ Decreasing unnecessary resource utilization within this population is a potential target for cost-effective, high-value care.^4–6^ Recent advances in microbiologic testing have made a more rapid clinical assessment and risk stratification possible in this patient population, with the increasing use of C-reactive protein, viral polymerase chain reaction (PCR) testing, and procalcitonin. Therefore, the cost and morbidity associated with prolonged hospitalization should be avoided in the low-risk patient.^7^ Although there is not yet a national consensus guideline for the diagnosis and management of febrile infants under 60 days of age, a recent study suggested that 3 laboratory data points may be used to risk-stratify these infants: urinalysis, absolute neutrophil count, and procalcitonin.^8^

The variability in the evaluation of febrile infants is well established. The management of these patients can vary based on the clinical setting (ie, outpatient office, emergency room, academic center, etc.).^9^ Infants presenting to the emergency room have more diagnostic testing performed and a higher likelihood of being admitted to the hospital as opposed to those patients being evaluated first in a primary care setting.^10^ Additionally, infants covered by state insurance programs have a higher likelihood of emergency room utilization.^11^ Therefore, we postulated that for our patient population (which is predominantly insured by state insurance programs), there was an opportunity to avoid excess resource utilization.

We participated in a multicenter Quality Improvement project through the American Academy of Pediatrics, titled “Reducing Variability in the Infant Sepsis Evaluation (REVISE).”^12^ REVISE required the participation of a pediatric emergency medicine provider and a pediatric hospital medicine provider at each site on the quality improvement team. This interdisciplinary team composition aimed to increase collaboration and align care practices based on a standard approach to risk stratification. REVISE participating sites could track various outcome measures ranging from antibiotic administration, chest x-ray utilization, and length of stay (LOS). Our SMART aim was to decrease the LOS for well-appearing febrile infants by 20% over 8 months from December 2016 to August 2017.

## METHODS

### Clinical Setting

The University of Illinois at Chicago Children’s Hospital is an academic children’s hospital within an adult hospital. Our pediatric emergency room is integrated into the adult emergency department—pediatric emergency medicine physicians, as well as general emergency medicine physicians, staff the pediatric emergency room. Our emergency department has an average of approximately 8,000 pediatric patient encounters annually, with an approximately 10% admission rate. General pediatricians and family medicine physicians staff our inpatient pediatric unit.

We participated in the REVISE collaborative through the American Academy of Pediatrics’ Value in Inpatient Pediatrics network from 2015 to 2018. The collaborative aimed to standardize the diagnostic evaluation and management approach by clinicians evaluating well-appearing febrile infants of ages 7–59 days at the participating sites. The REVISE collaborative offered our pediatric hospital medicine and pediatric emergency medicine faculty a framework to align their care practices.

### Study Population

All patients of ages 7–59 days presenting to the emergency department or admitted to the hospital, regardless of diagnosis, underwent manual chart review for study inclusion. This approach intended to avoid missing patients who were febrile at home, though not necessarily upon presentation. The University of Illinois at Chicago Institutional Review Board approved the study (IRB No. 2017-0058).

Exclusion criteria included the following: (1) patients who did not have a fever (either at home before or upon presentation), (2) ill-appearing patients at presentation, or (3) patients with significant congenital, genetic, or neurodevelopmental co-morbidities. These latter conditions put patients at higher baseline risk for acute bacterial infection (eg, severe combined immunodeficiency, or hydrocephalus). We also excluded patients who remained hospitalized from birth.

### Quality Improvement Team

We created a core quality improvement team consisting of 2 pediatric resident physicians, a pediatric hospitalist, and a pediatric emergency medicine physician. The team also consulted with an expert on quality improvement methodology within the pediatric department.

### Interventions

**Fig. 1.**
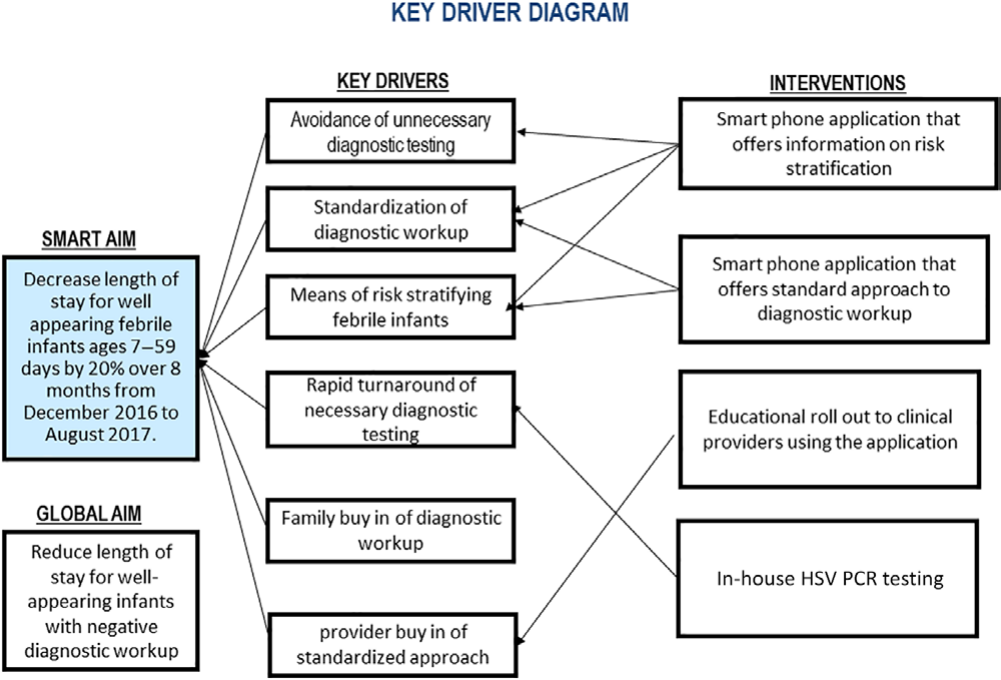
Aim and key driver diagram.

We used an electronic decision support tool to give clinicians real-time risk stratification and step-wise, patient-centered recommendations regarding diagnostic workup and management of the patients under their care. This smartphone application, CMPeDS, developed by the REVISE investigators, assessed infant risk based on historical and laboratory data.^13^ We postulated that excessive front-end diagnostic testing (and subsequent observation awaiting results) was a key driver in our LOS (Fig. 1). Therefore, our intervention promoting the use of a decision support smartphone application sought to help clinicians tailor the diagnostic workup to the patient’s characteristics, thereby avoiding unnecessary testing and decreasing LOS.

Our quality improvement team promoted smartphone application use as part of the clinical decision-making process via didactic lectures to pediatric residents, pediatric providers caring for pediatric inpatients, and emergency medicine faculty. We also used email reminders, inclusion in resident orientation materials, and the live demonstration of application use during inpatient rounds.

### Measures

#### Process Measure

The smartphone application was not integrated into our electronic health record (EHR) as part of this study. Therefore, we could not measure clinicians’ use of the smartphone application during patient care as a process measure.

#### Outcome Measure

Our outcome measure was the LOS in hours for all well-appearing patients of ages 7–59 days admitted to the Children’s Hospital of the University of Illinois, with a diagnosis of fever. LOS was measured from the first set of vital signs to the time of placement of discharge order.

#### Balancing Measure

Our balancing measure was the rate of readmission within 30 days for those well-appearing patients of ages 7–59 days admitted to the Children’s Hospital of the University of Illinois with a diagnosis of fever.

### Analysis

Per the parameters outlined by the REVISE collaborative, we performed monthly retrospective chart review from September 2015 to August 2016 to establish baseline data. For intervention data, we reviewed charts from December 2016 to August 2017, and from September 2017 to December 2018 for surveillance data.

Following our data collection, we engaged a local quality improvement expert to analyze the data. We used standard Statistical Process Control methods to analyze the data over time. Because the data are continuous with a fixed subgroup size of one, an XmR control chart was used, shown in Figure 3. Every admission that met inclusion criteria was included in our dataset. Mean and control limits were calculated from the pre-intervention data points. Subsequent data points were analyzed using standard Statistical Process Control rules for special cause. The upper and lower control limits of the mean are defined as three standard deviations above and below the mean.

**Fig. 2.**
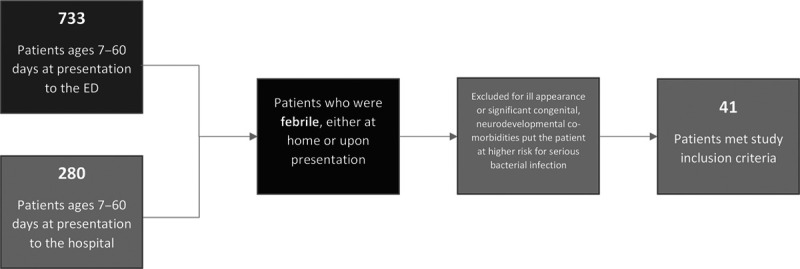
Flow diagram of patient selection based on study inclusion/exclusion criteria. ED, emergency department.

**Fig. 3.**
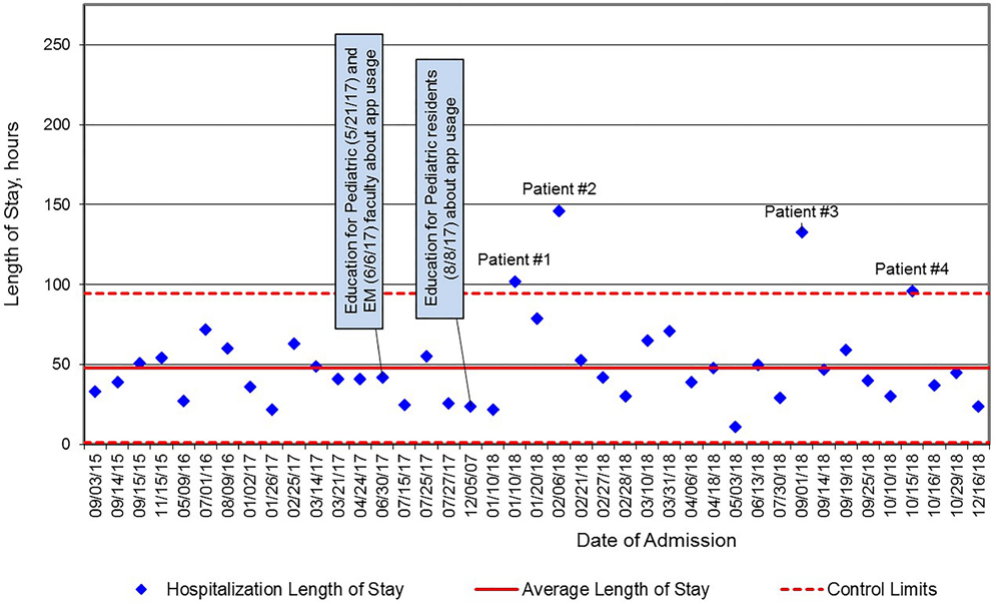
Length of stay of consecutive hospitalizations for well-appearing infants with fever, XmR chart September 2015 through December 2018.

## RESULTS

The team screened a total of 1,013 participants for inclusion in the study. Figure 2 demonstrates the total number of charts reviewed and subsequent patients who met inclusion criteria for our study, that is, well-appearing infants with a fever hospitalized for further evaluation. Overall, 41 infants met the inclusion criteria. Of the 41 well-appearing febrile infants between 7 and 59 days of age hospitalized during the study period at our hospital, 78% of those admissions originated from our emergency department, with 22% admitted directly from either an outpatient physician’s office or an outside emergency department. The mean LOS was 48.0 hours during the baseline period, with upper and lower control limits of 94.5 and 1.45 hours, respectively. Aside from the 4 data points outside the upper control limit, which we annotated as patients No. 1–4 and will discuss further, none of the other data met the criteria for special cause (Fig. 3). Therefore, recalculation of the mean and control limits was not done. However, we have annotated in Figure 3, the 4 individual cases whose LOS fell outside the upper control limit of 94.5 hours of our mean LOS. The mean LOS for patients 1–4 is 119.3 hours. The mean LOS without the patients 1–4 is 42.7 hours (*P* < 0.0001, using unpaired student’s *t* test, online calculator). There were no emergency room or hospital readmissions (balancing measures) within 30 days among study participants during the study period.

## DISCUSSION

Our results suggest that our current system has a well-defined mean LOS of 48.0 hours for well-appearing febrile infants of ages 7–59 days. We did not lower our mean LOS despite our intervention to standardize the diagnostic evaluation across clinical settings. The 4 individual cases whose LOS fell outside the upper control limits of our mean warrant further discussion. Both patients 1 and 2 were males found to have urinary tract infections. Patient 2 also had bacteremia. Both patients required intravenous antibiotic therapy until culture data was available to de-escalate to oral antibiotics. Patients 3 and 4 were both diagnosed with aseptic meningitis. In case 3, the inpatient team was suspicious of herpes simplex virus (HSV) meningitis. Therefore, they were awaiting HSV PCR test results from the cerebrospinal fluid to determine the ongoing clinical need for acyclovir. In case 4, the parents had not initially consented to lumbar puncture. Once the lumbar puncture was obtained 36 hours into the hospitalization, the enteroviral PCR was positive in the cerebrospinal fluid, solidifying the diagnosis. Our results suggest that a standardized approach to the diagnostic evaluation can help identify those infants with urinary tract infection, bacteremia, or meningitis.

Byington et al^14^ suggest that LOS could be targeted to less than 24 hours for low-risk patients without adverse outcomes. The authors of this study define low-risk infants as those without urinary tract infection, bacteremia, and meningitis. This definition is different from that used for the inclusion criteria of our study. Our study included all well-appearing infants at the initial presentation. It was not until after further diagnostic evaluation that they could be deemed as having urinary tract infection, bacteremia, or meningitis. Our mean LOS for patients who met our inclusion criteria (excluding patients No. 1–4 as outliers with significant disease burden as high risk) was 42.7 hours. Our future efforts to decrease LOS to these study authors’ proposed length should focus on efforts after standardized diagnostic evaluation to exclude urinary tract infection, bacteremia, or meningitis. This exclusion should be clarified when creating a SMART aim in the future.

Additionally, REVISE study authors^12^ excluded infants diagnosed with urinary tract infection, bacteremia, or meningitis from LOS reduction targets. REVISE study authors^12^ considered “non–low risk” patients to be those of age <31 days, with abnormal findings on laboratory workup, with relevant past medical history, or with required admission for social reasons. For this group, they described a target LOS of 42 hours. For all other low-risk infants, they considered a target LOS of <30 hours. Well-appearing, febrile infants not diagnosed with urinary tract infection, bacteremia, or meningitis comprised approximately 90% of the infants in our study.

To achieve a decrease in the LOS for febrile infants identified with urinary tract infection, bacteremia, or meningitis, we will need to address other key drivers. These key drivers include in-hospital access to HSV PCR testing (rather than reliance on send-out testing) and increasing family buy-in for the necessary diagnostic evaluation (including invasive testing such as a lumbar puncture) at presentation. However, given the variation in diagnosis represented by infants in this group, an LOS metric for this group of patients is not uniform.

We promoted the use of a decision support tool to standardize the diagnostic approach to febrile infants. The purpose of the decision support tool, CMPeDS, was introduced to both faculty and learners in a primarily education-focused manner (ie, didactic teaching sessions, inclusion in orientation materials, etc.). Quality improvement science acknowledges that education-focused interventions are not the most effective means of bringing about sustained, significant change. Therefore, our future work should encompass more than education-based interventions. We should advocate for systems-based processes, such as embedding the decision support tool into our EHR. If the decision support tool was embedded into our EHR, we could measure its use by clinicians as a process measure. This change would allow awareness as to whether or not clinicians are using the tool we promoted. However, this tool would have to be integrated into clinical workflow thoughtfully. The simple presence of a decision support tool in the EHR alone is also not sufficient to change clinical behavior.

The composition of our quality improvement team was critical to this project. Our quality improvement team included faculty from both hospital medicine and pediatric emergency medicine. The interdisciplinary structure of the quality improvement team enabled the Departments of Pediatrics and Emergency Medicine to work together to create a shared mental model around the diagnostic approach to the well-appearing febrile infant. Additionally, pediatric residents participated as members of our quality improvement team. Our pediatric residents work in both our emergency department and inpatient units. Therefore, they were ideal members of our clinical workforce to disseminate information about the smartphone application for treatment decisions.

### Limitations

Our small sample size precludes definitive conclusions that are broadly generalizable. However, this size was sufficient to note the marked difference in LOS between patients with and without urinary tract infection, bacteremia, or meningitis, requiring different interventions to reduce LOS. Second, the smartphone application was not embedded within our EHR. Therefore, despite our promotion of its use, we could not measure the actual use of the decision support tool by clinicians as a process measure during our study. Third, our Children’s Hospital is located in an urban metropolis with 3 other large academic medical centers within the city of Chicago. Because we are using our local EHR data, not payor data, readmission rates include only return visits to our institution and may miss patients who re-present to other facilities.

## CONCLUSIONS

A clinical decision support tool may help to standardize the diagnostic and management approach to the well-appearing febrile infant across clinical settings. This standardization occurs by consistent risk-stratification using patient-centered historical and laboratory factors. This standardized approach may reduce the variation in clinical decision-making. This approach may effectively identify infants with urinary tract infection, bacteremia, or meningitis. In our study, infants with urinary tract infection, bacteremia, and meningitis were outliers to our mean LOS. Therefore, future attempts to decrease the LOS may require different interventions for those infants with urinary tract infections, bacteremia, and meningitis in contrast to those without these diagnoses.

## DISCLOSURE

Dr. Antoon was supported by the National Heart, Lung, and Blood Institute of the National Institutes of Health under Award Number K12 HL137943. The content is solely the responsibility of the authors and does not necessarily represent the official views of the National Institutes of Health. The other authors have no financial interest to declare in relation to the content of this article.

## ACKNOWLEDGMENTS

The authors acknowledge the American Academy of Pediatrics VIP Network for REVISE study and The Children’s Mercy Hospital, Kansas City, MO 2016, for CMPeDs App.
